# Shrinking Weibel‐Palade bodies prevents high platelet recruitment in assays using thrombotic thrombocytopenic purpura plasma

**DOI:** 10.1002/rth2.12626

**Published:** 2021-12-07

**Authors:** Francesca Patella, Chiara Vendramin, Oscar Charles, Marie A. Scully, Daniel F. Cutler

**Affiliations:** ^1^ MRC Laboratory for Molecular Cell Biology University College London London UK; ^2^ Kinomica Alderley Park Alderley Edge Macclesfield UK; ^3^ Haemostasis Research Unit University College London London UK

**Keywords:** blood platelets, fluvastatin, human, thrombotic thrombocytopenic purpura, von Willebrand factor, Weibel‐Palade bodies

## Abstract

**Background:**

Thrombotic thrombocytopenic purpura (TTP), caused by a genetic or autoimmune‐driven lack of ADAMTS‐13 activity, leads to high levels of the ultra‐large von Willebrand factor (VWF) multimers produced by endothelial cells, causing excess platelet recruitment into forming thrombi, often with mortal consequences. Treatments include plasma infusion or replacement to restore ADAMTS‐13 activity, or prevention of platelet recruitment to VWF.

**Objectives:**

We tested a different approach, exploiting the unique cell biology of the endothelium. Upon activation, the VWF released by exocytosis of Weibel‐Palade bodies (WPBs), transiently anchored to the cell surface, unfurls as strings into flowing plasma, recruiting platelets. Using plasma from patients with TTP increases platelet recruitment to the surface of cultured endothelial cells under flow. WPBs are uniquely plastic, and shortening WPBs dramatically reduces VWF string lengths and the recruitment of platelets. We wished to test whether the TTP plasma‐driven increase in platelet recruitment would be countered by reducing formation of the longest WPBs that release longer strings.

**Methods:**

Endothelial cells grown in flow chambers were treated with fluvastatin, one of 37 drugs shown to shorten WPBs, then activated under flow in the presence of platelets and plasma of either controls or patients with TTP.

**Result:**

We found that the dramatic increase in platelet recruitment caused by TTP plasma is entirely countered by treatment with fluvastatin, shortening the WPBs.

**Conclusions:**

This potential approach of ameliorating the endothelial contribution to thrombotic risk by intervening far upstream of hemostasis might prove a useful adjunct to more conventional and direct therapies.
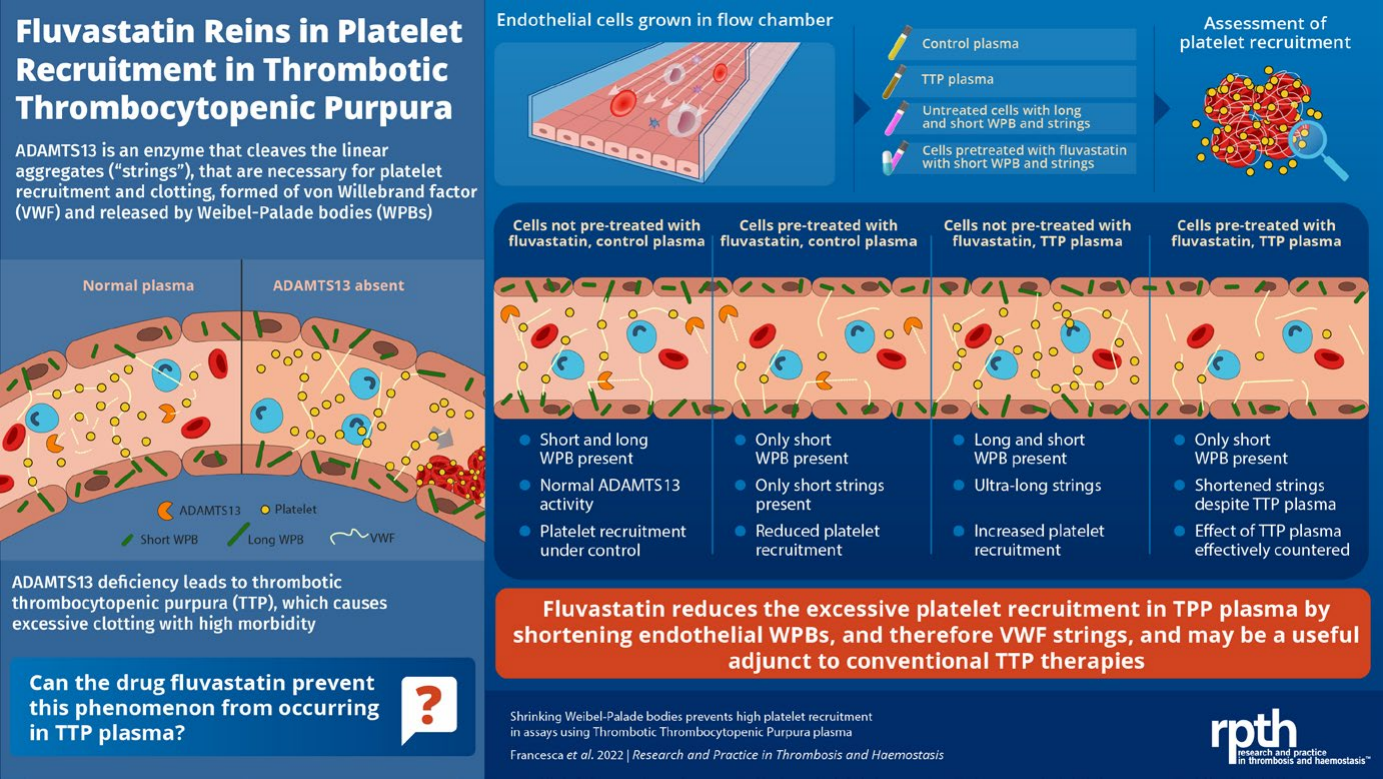

**Essentials**

- The excess ultra‐large VWF of thrombotic thrombocytopenic purpura (TTP) causes high morbidity.
- Plasma of TTP patients causes high VWF and platelet recruitment to activated endothelium under flow.
- Fluvastatin pretreatment of the endothelial cells completely ablates this effect of plasma of TTP patients.
- These data suggest that fluvastatin might be a useful adjunct therapy in TTP.

## 1 INTRODUCTION

Thrombotic thrombocytopenic purpura (TTP) is a severe disease resulting in multiorgan failure and, untreated, has a mortality of >90%. TTP arises from a severe deficiency of ADAMTS‐13 activity,^1^, ^2^, ^3^ a circulating plasma metalloprotease, the only known substrate for which is von Willebrand factor (VWF).

VWF undergoes a complex biosynthesis in endothelial cells, culminating in the formation of tubules of multiple coiled ultra‐large (UL‐VWF) multimers that drive the formation of the rod‐shaped endothelial secretory granules, Weibel‐Palade bodies (WPBs).^4^, ^5^, ^6^ Endothelial activation drives WPB exocytosis to release their content into the plasma, where flow unfurls the tubular coils of UL‐VWF into long strings anchored to the endothelial surface by an unknown mechanism.^7^ The transient strings are highly active in platelet recruitment and are incorporated into primary hemostatic structures or are cleaved by ADAMTS‐13 into the smaller VWF multimers seen in plasma.^8^ We recently showed that the lengths of VWF strings (but not VWF’s multimeric state) change with the length (ie, size) of WPBs; that WPBs are formed in a range of sizes from 0.5 to 5 μm and that their size is affected by physiological cues, or by drugs that affect the modulators of WPB size (Golgi linkage, secretory trafficking rate, and VWF expression level).^9^, ^10^, ^11^, ^12^

In TTP, the lack (either genetic or immune mediated) of functional ADAMTS‐13 precludes proteolysis of the just‐secreted UL‐VWF multimers. UL‐VWF incorporated into relatively stable strings then forms platelet/plasma VWF hemostatic structures or escapes into circulating plasma. With UL‐VWF persisting, excess platelet recruitment to strings or into thrombi then occurs.^13^, ^14^, ^15^, ^16^, ^17^ We describe here a potential approach toward ameliorating congenital TTP (cTTP) that focuses on modulating endothelial cell function. We show, in these in vitro proof‐of‐concept experiments, that we can repurpose a representative of well‐understood drugs, statins, that we previously identified as able to decrease WPB size, thus decreasing string lengths, to reduce the platelet recruitment under flow in vitro. Fluvastatin decreases the number and length of VWF strings by reprogramming the size of newly forming WPB in endothelial cells in vitro,^10^, ^12^ by an “organelle‐directed medicine” approach,^18^ producing results consistent with their known anti‐inflammatory and anticoagulant^19^, ^20^ effects in vivo. Our proof‐of‐concept experiments comparing plasma from controls and patients with cTTP show that overcoming ADAMTS‐13 deficiency and ablating excess platelet adhesion to VWF in cTTP might be possible using fluvastatin.

## 2 MATERIALS AND METHODS

### 2.1 Patient selection

Nine patient episodes with cTTP under the care of a single tertiary center were identified from the UK TTP Registry (Medical Research Ethics Committee Numbers 08/H0810/54 and 08/H0716/72) and characterized here.^21^ cTTP was defined as patients with ADAMTS‐13 activity <10 IU/dL, no evidence of anti–ADAMTS‐13 IgG antibodies and the identification of an ADAMTS‐13 mutation causing cTTP. The female patients were diagnosed in adulthood (median age, 26), the male in infancy, aged 6. Two of the six women (patient 1 and patient 2) were pregnant at the time of sampling and were also resampled after pregnancy, respectively, 6 and 7 months after delivery. One patient (patient 3) was a new presentation, diagnosed immediately postpartum and sampled 1 month after delivery. All patients were routinely monitored with laboratory parameters in normal range not suggestive of acute or subacute disease and received regular plasma infusion therapy, typically 10 mL/kg Octaplas (Octapharmal Lachen, Switzerland). One patient was treated with intermediate purity factor VIII concentrate (BPL‐8Y), ≈15 IU/kg (see Table 1). For all cases, the following parameters were included: demographic information, full blood count, ADAMTS‐13 activity, ADAMTS‐13 antigen, VWF activity, VWF antigen, flow‐based assay parameters.

**TABLE 1:** Laboratory and Clinical Characteristics of 7 patients with congenital TTP. Patient 1 and Patient 2 provided samples during both pregnancy and post‐pregnancy (1 p.p. and 2 p.p.)

Patients	Sex	Age at time of sampling, y	Age at diagnosis, y	VWF ag (normal range: 0.5‐1.60 IU/mL)	VWF ac (normal range: 0.5‐1.87 IU/mL)	AD13 ac (normal range: 64‐132 IU/dL)	AD13 ag (normal range: 74.4‐134.4%)	PLT (normal range: 150‐400 × 10^9^/L)	LDH (normal range: 135‐214 IU/L)	Diagnosis	Genetic mutation	Ongoing treatment at time of sampling	Other
Pooled normal plasma (Cryocheck)	NA	NA	NA	0.8	0.8	91	110	NA	NA	NA	NA	NA	NA
1	F	43	39	2.8	2.8	<5	8.1	266	164	Congenital TTP, pregnant at 32 weeks gestation at time of sampling	Exon 24 R1060W Hom, Exon 24 A1033T Hom	800 ml plasma twice a week until delivery planned for 27/06/18, Aspirin 75 mg daily, Fragmin 5000 units daily	
1 p.p	F	43	39	2.3	2.7	<5	3	300	NA	Congenital TTP, 6 mo after delivery	Exon 24 R1060W Hom, Exon 24 A1033T Hom	1000 mL plasma every 2 wks	
2	F	29	17	2.4	3	<5	1.7	242	167	Congenital TTP, pregnant at 34 wks gestation at time of sampling	Exon 29: 4143InsA	800 mL plasma weekly, Aspirin 75 mg daily, Fragmin 5000 units daily	
2 p.p.	F	29	17	1	1.4	<5	1	326	NA	Congenital TTP, 7 mo after delivery	Exon 29: 4143InsA	600 mL plasma every 2 wks	
3	F	30	30	1.9	2.5	<5	5.5	289	171	Congenital TTP (new presentation, immediate postpartum – diagnosed in June 2018)	Exon 24 R1060W Het, Exon 24 A1033T Het	600 mL plasma every 2 wks Aspirin 75 mg daily (started at beginning of July 2018)	Thalassaemia trait; Sample collected 1‐mo postpartum period
4	F	35	21	0.9	1	<5	6	288	189	Congenital TTP	Exon 24 R1060W Het, Exon 28 c.3015delA	800 ml plasma weekly, Aspirin 75 mg daily	
5	F	70	21	2.9	3.1	<5	3	138	NA	Congenital TTP	Exon 7 c.719_724del Het, Exon 17 A690T Het	600 ml plasma every 3 wks	
6	F	44	37	1.7	2.6	<5	4	411	202	Congenital TTP	Exon 10 c.386delG, Exon 24 R1060W Het, Exon 16 P618A Het	800 mL plasma every 2 wks	
7	M	37	6	2.3	3	<5	1	189	179	Congenital TTP	Exon 7 G236C Het, Exon 24 W1016X Het	2000 units of BPL8Y weekly with low‐molecular‐weight heparin prophylactic cover, Aspirin 75 mg daily	

Abbreviations: Fragmin, low‐molecular‐weight heparin; N/A, not applicable; p.p., post pregnancy.

### 2.2 Patients’ plasma collection

Citrated plasma was collected 30 minutes before prophylactic treatment, when patients were not experiencing an acute cTTP event, with normal complete blood counts. A double‐centrifugation method was used for all the samples. Patients’ plasma samples were processed within 4 hours of venesection and subsequently frozen at −80°C. CRYOchek (cat. no. CCN‐15; Precision BioLogic, Dartmouth, NS, Canada) was used as control pooled normal plasma.

### 2.3 ADAMTS‐13 and VWF assays

ADAMTS‐13 activity in patients’ plasma was measured using the FRETS VWF73 method (normal range, 60‐146 IU/dL)^22^ and ADAMTS‐13 antigen (normal range, 74%‐134%) was quantified by ELISA.^23^ VWF activity (normal range, 0.5–1.87 IU/dL) and VWF antigen (normal range, 0.50–1.60 IU/mL) were performed using a standard automated immuno‐turbimetric assay in a Sysmex CS2100i analyzer (Sysmex Corporation, Kobe, Japan) with a Siemens kit (VWF:Ag and INNOVANCE VWF:Ac; Siemens Healthcare Diagnostics, Deerfield, IL, USA).^24^

### 2.4 Cell culture

Human umbilical vein endothelial cells (HUVECs) pooled from multiple donors from PromoCell (Heidelberg, Germany) were used within the fourth passage. Cells were cultured in M199 (Thermo Fisher Scientific, Waltham, MA, USA) with 20% foetal bovine serum (Labtech, Heathfield, UK), 30 μg/mLl endothelial cell growth supplement from bovine neural tissue (Sigma‐Aldrich, St. Louis, MO, USA) and 10 U/mL heparin (Sigma‐Aldrich). Forty thousand cells were seeded on gelatin‐coated Ibidi μ‐slides providing a rectangular channel (Thistle Scientific, Glasgow, Scotland). The day after, upon reaching confluency, they were treated with 2 µM fluvastatin (Sigma‐Aldrich) in dimethylsulfoxide (DMSO) or with DMSO as a control.

### 2.5 Flow assay

After 24 hours of fluvastatin or control treatment, cells seeded in the Ibidi μ‐slides were washed with complete medium plus 15 mM 4‐(2‐hydroxyethyl)‐1‐piperazineethanesulfonic acid (HEPES; Thermo Fisher Scientific). The slides were attached to a pump system (Harvard Apparatus, Holliston, MA, USA) and maintained at 37°C. A constant wall shear stress of 2.5 dynes/cm^2^ (corresponding to a flow rate of 1.4 mL/min) was maintained throughout the experiments. All added solutions were prewarmed at 37°C before perfusion. The cells were initially perfused with Hank’s balanced salt solution (HBBS; Life Technologies, Rockville, MD, USA) supplemented with 0.2% bovine serum albumin (BSA), Ca^2+^, and Mg^2+^ for 2 minutes, then with HBBS containing 100 µmol L^−1^ of histamine (cat. No. ALX‐550–132; Enzo Life Sciences, Farmingdale, NY, USA) for a further 3 minutes to stimulate the exocytosis of WPBs. This was followed by perfusion with plasma from patients with TTP or control plasma supplemented with histamine and platelets (spared pooled platelets from the Blood Transfusion service, at a suspension of 10^8^/mL) for an additional minute. Finally, after a final perfusion wash with HBSS, cells were fixed with 4% formaldehyde in phosphate buffered saline (PBS) for 2 minutes and then under reduced flow (0.7 mL/min) for 2 additional minutes. Flow was then stopped, and cells were left to fix for a further 11 minutes under static conditions, washed with PBS, processed for immunofluorescence, and imaged (see below). The general workflow used one control run for each run of experimental samples. Each set thus included one control +/− fluvastatin, and two to four TTP samples +/− fluvastatin, thus conserving samples and cells while limiting environmental drift between experimental examples and controls on any given session as much as possible.

### 2.6 Immunocytochemistry

After the flow assay, fixed HUVECs and platelets in μ‐slides were blocked with 5% BSA diluted in PBS for 20 minutes. Samples were subsequently incubated with primary antibodies, washed in PBS, then incubated in secondary antibodies. All antibodies were diluted in 1% BSA in PBS and incubated for 1 hour. Antibodies used anti‐VWF (1:1000) (DAKO; cat. No A00A2, Agilent Technologies LDA UK Ltd), anti‐CD41‐FITC (1:200) (Millipore, Burlington, MA, USA; clone 5B12, FCMAB195F). Secondary antibodies: AlexaFluor 488, 564 (Life Technologies) at 1:500 dilution. Hoechst 33342 (Life Technologies) was used to counterstain nuclei (1: 10 000).

### 2.7 Confocal microscopy

Imaging was performed using a spinning‐disc Ultraview Vox confocal microscope with a 20× objective and 1.5× tube lens. A tiled image panel was taken comprising 36 images in a 3 × 12 grid with 30 μm z depth.

### 2.8 Image analysis

VWF and platelet adherence to the HUVEC monolayer was quantified by image analysis in Fiji (ImageJ). Each field of view (35 images per sample) was assessed independently. The z‐stacked images were transformed into a maximum intensity Z‐projection, and the fluorescence signal of VWF and CD41 then thresholded and converted into binary values. The area of each image occupied by VWF particles or platelets was measured using the “Analyse particle” function within ImageJ. In order to segment VWF strings in images taken under laminar flow, we developed a Python script.^25^ It implements a set of convolution matrices to remove nonstring VWF agglomerates and highlight regions of discrete contiguous signal aligned with flow direction. We used a threshold to produce a binary image and measured the properties of these unique regions.

### 2.9 Statistical analysis

Statistical analyses were performed using Prism software version 7 (GraphPad Software, La Jolla, CA, USA). The tests used to assess statistical significance are indicated in the figure legends.

## 3 RESULTS

Plasma was obtained from seven patients with congenital TTP; six women and one man (median age, 37; range, 29‐70 years). The level of ADAMTS‐13 activity at the time of sample collection, that is, immediately before plasma infusion therapy, was <5 IU/dL (normal range, 64‐132 IU/dL), whereas VWF antigen and activity levels were both above the normal range for all patients, except for patient 4 and patient 2 (postpregnancy; see Table 1).

To test the effect of statins on platelet and plasma VWF recruitment in the presence of plasma from pooled controls or cTTP patients, we treated human umbilical vein endothelial cells (HUVECs) with 2 μM fluvastatin or vehicle (DMSO) for 24 hours, a time frame allowing the natural turnover of WPBs to allow replacement by organelles formed in the presence of the drug. By differentially reducing the formation of longer granules, fluvastatin will shift the intracellular population of WPB toward a shorter size distribution.^10^, ^12^ We then analyzed platelet and/or plasma VWF recruitment to the surface of control or drug‐treated cells under flow. We used a protocol designed to simplify the interpretation of our data, that could produce a clear effect of statins (or other WPB size‐altering molecules) on both string production and platelet recruitment.^9^, ^10^ Confluent HUVECs grown in a flow chamber containing control or fluvastatin‐shortened WPB were washed under flow (and kept under continuous flow until after initial fixation at the end of the experiment) into a simple salt solution, then activated with histamine for 3 minutes. We chose histamine as a simple, effective, widely used physiological agonist that produces a rapid exocytic response, giving a pulse of VWF release that lasts about 5 minutes, and that drives unbiased exocytosis of all sizes of WPBs.^26^ At 3 minutes, that is, during the peak response to agonist, either patient or commercially sourced pooled control plasma plus histamine and control platelets was added, followed after 60 seconds by washing, fixation, and immunostaining. After confocal imaging, the number and lengths of VWF strings and the number of platelets adhering to the endothelial monolayer in these images were automatically quantified.

**FIGURE 1:**
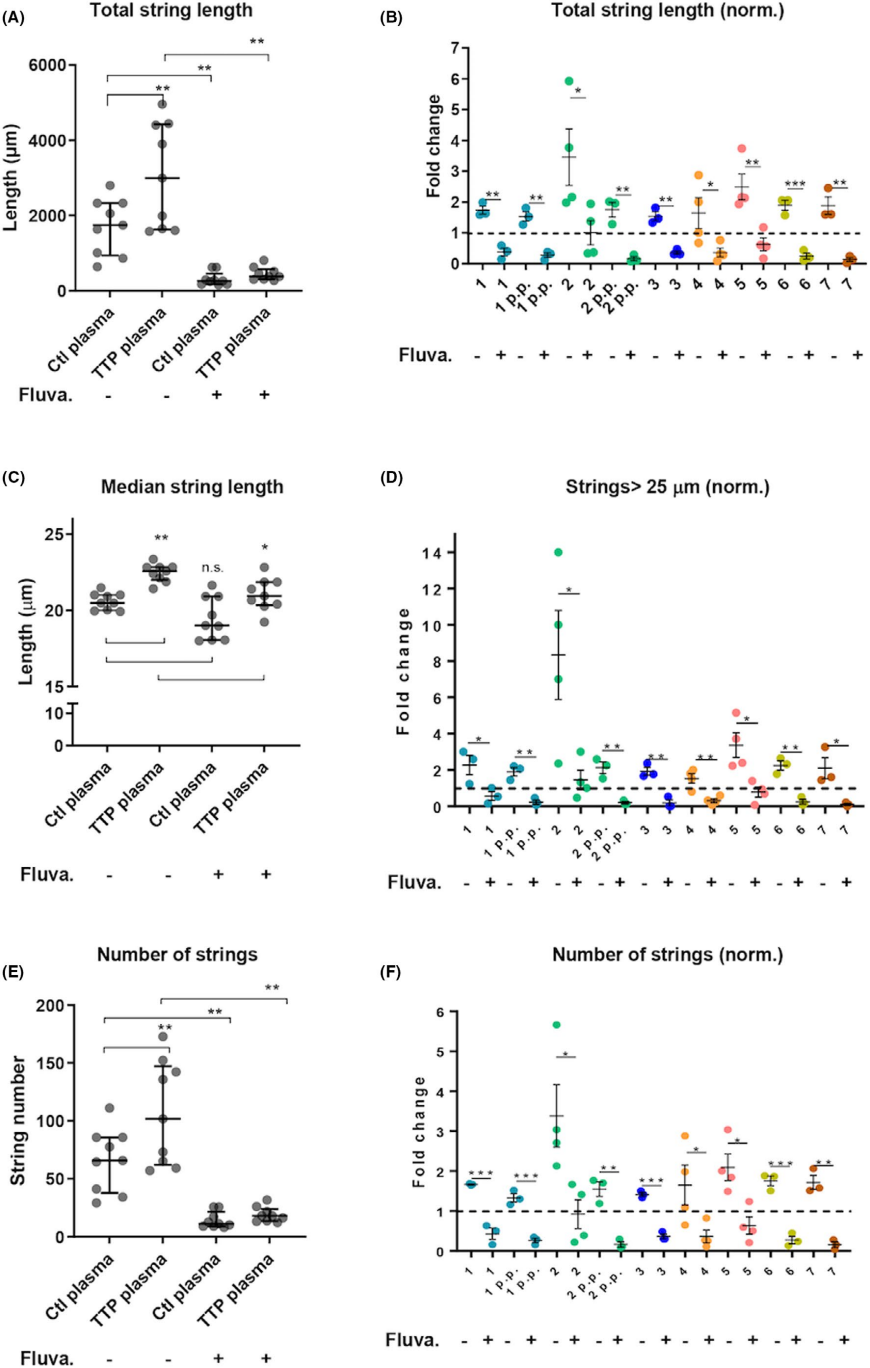
Fluvastatin decreases total cumulative VWF string length in the presence of TTP plasma. Endothelial cells were treated with 2 μM fluvastatin or DMSO for 24 hours before flow assays were performed. Cells were stimulated with histamine under flow and then superfused with platelets added to plasma from control or TTP patients. After fixation and immunofluoresce with anti‐VWF and anti‐CD41 antibodies, VWF strings and platelets were quantified. A, Sum of all string lengths. Each dot represents the mean of the total string length measured over 3 experiments. N = 9 patients’ plasma samples or their controls. Median and interquartile ranges are shown. Paired samples Wilcoxon test, **p < 0.005. B, Sum of string lengths for each patient, normalised to their corresponding plasma control (dotted line). N = 3 independent experiments. Mean and SEM are shown. Unpaired *t* test, *P < .05, **P < .005, ***P < .001. C, Median string length. Each dot is the median string length measured over 3 experiments, n = 9 patient plasma or their corresponding controls. Median and interquartile ranges are shown. Paired samples Wilcoxon test, **P < .005, *P < .05. D, Number of “long” strings (>25 µm) measured for each patient’s plasma normalized to the corresponding plasma control (dotted line). N = 3 independent experiments. Mean and SEM are shown. Unpaired *t* test, **P < .005, *P < .05. E, Number of all strings normalized for the corresponding plasma control. Each dot is the average number measured over 3 experiments, n = 9 patients’ plasma or their corresponding controls. Median and interquartile ranges are shown. Paired samples Wilcoxon test, **P < .005. F, Number of strings for each patient, normalised to the corresponding plasma control (dotted line). N = 3 independent experiments. Mean and SEM are shown. Unpaired *t* test, ***P < 0.001. DMSO, dimethylsulfoxide; SEM, standard error of the mean; TTP; thrombotic thrombocytopenic purpura; VWF, von Willebrand factor

Our analyses of string length and number found that the total cumulative string length increased almost twofold in the presence of patient as compared to control plasma (Figure 1A, B). Increases ranged from 1.47‐fold in patient 1 after pregnancy to 3.89‐fold in patient 2, consistent with strings being more stable in the absence of ADAMTS‐13.^13^ We also confirmed that a 24‐hour pretreatment with fluvastatin decreased the total cumulative length to below no‐statin levels in assays using both control and patient plasma (Figure 1A, B). The median string length (Figure 1C) was also significantly longer in strings formed with TTP plasma, but did not fall as far as the total string length when treated with fluvastatin, suggesting that we are differentially suppressing the very longest, and most functionally active^10^ fraction of strings. We also show a simplified metric; strings longer than 25 μm (Figure 1D), since this differentiating point in string length provides a clear relationship to functional activity. Fluvastatin treatment significantly reduced the number of strings longer than 25 μm in all patients.

Not only the length but also the number of strings is increased in the presence of patient plasma (Figure 1E, F), presumably due to the increased stability offered by absence of ADAMTS‐13 in the plasma, plus potentially also recruitment of the UL‐VWF present in patient plasma to further stabilize as well as extending the existing strings. The number of strings is also reduced by fluvastatin treatment to below control levels irrespective of the source of plasma present. Finally, there is a rough relationship between which patients’ plasma was used in the assay, the cumulative length, the fraction of strings longer than 25 μm and the number of strings seen in the presence or absence of fluvastatin treatment. This encourages us to believe that our assay is capable of discriminating between the small changes (shown in Table 1) in the plasma content between patients, and thus likely to be of utility.

**FIGURE 2:**
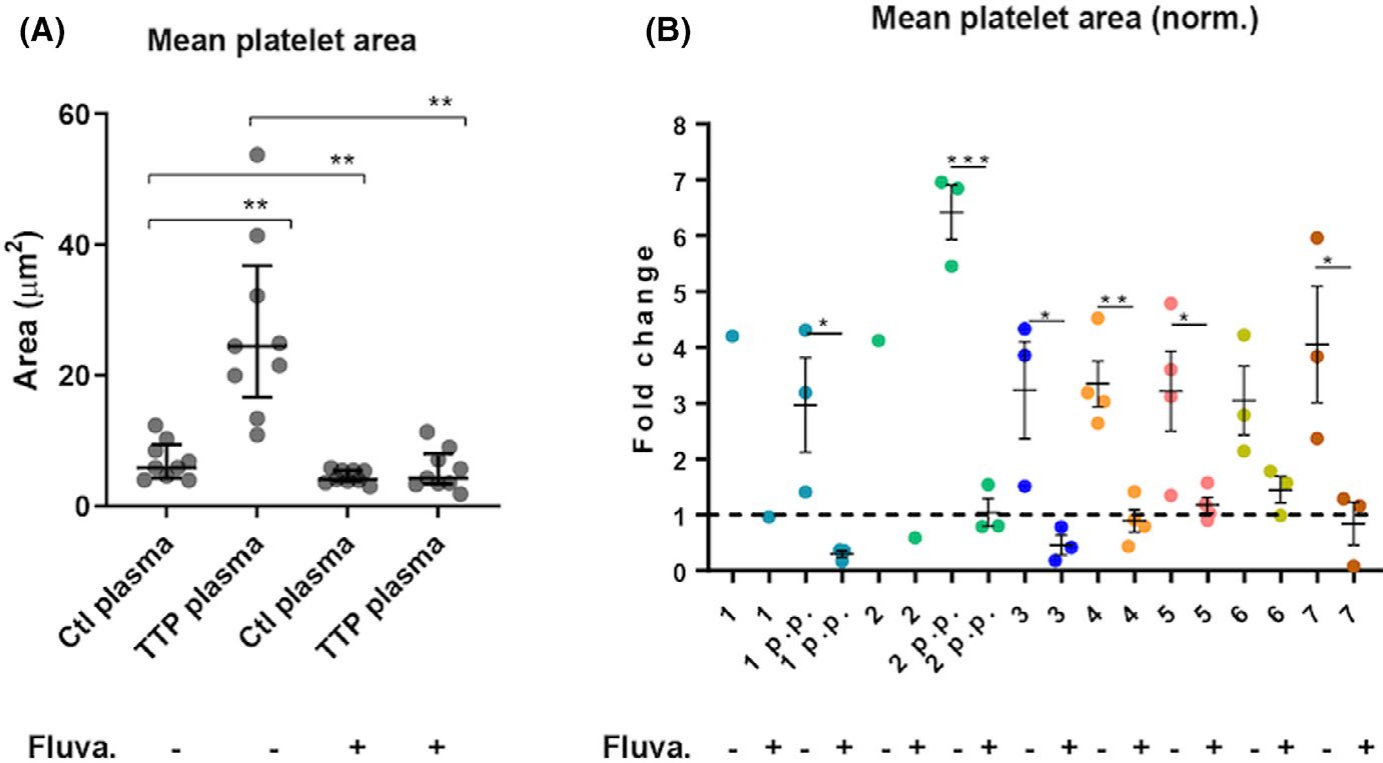
Fluvastatin decreases platelet recruitment on endothelial monolayer superfused with TTP plasma. Endothelial cells were pretreated with 2 μM fluvastatin or DMSO for 24 hours, then flow assays were performed. Cells were stimulated with histamine under flow and then superfused with platelets added to plasma from control or TTP people. After fixation and immunofluorescence labelling with anti‐CD41 antibody, platelets were quantified. A, Mean area covered by platelets adhered on the endothelial cells. Each dot represents the mean area covered by platelets per FOV, averaged over 3 experiments, n = 9 patients’ plasma or their correspondent controls. Median and interquartile ranges are shown. Paired sample Wilcoxon test, **P < .005. B, Area covered by platelets adhered on the endothelial cells measured for each patient’s plasma, normalized for the correspondent plasma control. N = 3 independent experiments. Mean and SEM are shown. *t* test, ***P < .001, **P < .005, *p < .05. DMSO, dimethylsulfoxide; SEM, standard error of the mean; TTP; thrombotic thrombocytopenic purpura

We also analyzed comparative platelet recruitment. We found an average 3.87‐fold increase in the area covered by platelets recruited to the untreated endothelial monolayer incubated with patient plasma as compared to control plasma (Figure 2A), but platelet recruitment was significantly inhibited by fluvastatin not only in the presence of control but even more dramatically in the presence of patient plasma in flow assays (Figure 2A, B).

**FIGURE 3:**
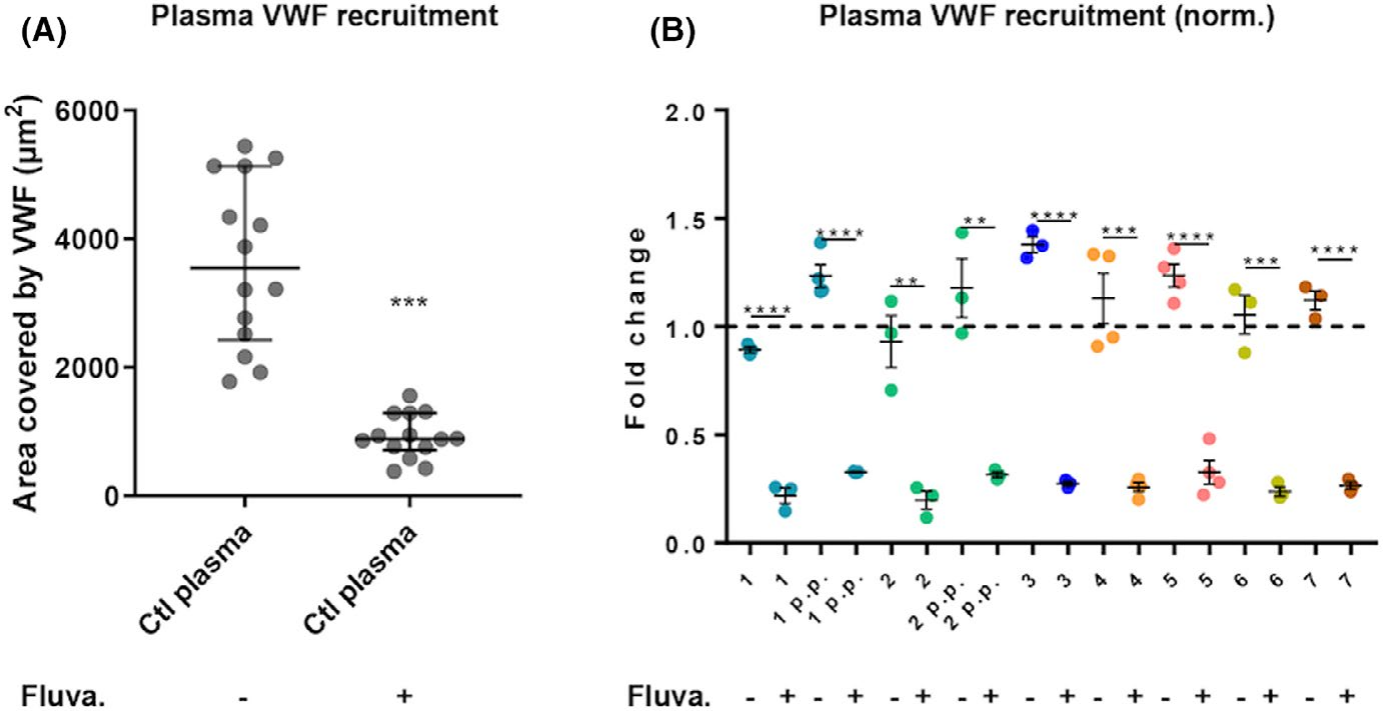
Fluvastatin decreases the recruitment of plasma VWF in the presence of TTP plasma. Endothelial cells were treated or not with 2 μM fluvastatin for 24 hours, then flow assays were performed. Cells were stimulated with histamine under flow and then superfused with plasma from pooled controls or individual TTP patients. After fixation and immunofluoresce with anti‐VWF antibody, the area covered by VWF on the endothelial monolayer was quantified. A, Area of endothelial cell surface covered by VFW upon infusion of control plasma and pretreatment with fluvastatin. Paired samples Wilcoxon test, ***P < .001. B, Area of endothelial cell surface covered by VFW, measured for each patient’s plasma, normalized for the correspondent plasma control. N = 3 independent experiments. Mean and SEM are shown. Unpaired *t* test, ***P < 0.001, **P < 0.005. SEM, standard error of the mean; TTP; thrombotic thrombocytopenic purpura; VWF, von Willebrand factor

Finally, fluvastatin also caused a dramatic (an average of 3.71‐fold) fall in total VWF present on the endothelial surface, a measure combining not only the VWF present in strings, but also exocytosed, surface‐retained VWF that has not formed strings, plus plasma VWF recruited to the cell surface. In vivo these could all potentially contribute to sustained platelet adhesion and thrombus formation (Figure 3A, B).^27^

We conclude that these mutually supportive data reinforce the hypothesis that fluvastatin decreases the endothelial capacity for plasma VWF and platelet recruitment, significantly diminishing its potential prohemostatic capacity and effectively countering at least these parameters caused by the loss of a normal level of ADAMTS‐13 function as observed in the plasma of patients with TTP.

## 4 DISCUSSION

In this study, we explored the potential use of reprogramming endothelial cells into producing shorter WPBs that contain VWF with lowered platelet/plasma VWF‐recruiting capacity. We have chosen congenital TTP as a simple genetic disorder causing massively increased thrombotic risk and morbidity, but this approach could potentially be extended to the study of immuno‐mediated TTP, as it acts by circumventing the lack of ADAMTS‐13 functionality. Current therapies for TTP include plasma infusion and plasma exchange to replenish the level of ADAMTS‐13 reducing UL‐VWF multimers. For congenital TTP, early diagnosis is possible, allowing for plasma‐replacement prophylaxis to begin. However, if dosing and uptake is not optimal, a high risk of acute episodes with ischemic stroke and other consequences exist, as well as nonovert symptoms like lethargy, headache, mental disorders, and abdominal pain.^21^ The disadvantages of such therapies include frequent hospital visits (every 1‐2 weeks), the possibility of fluid overload and of developing an allergic reaction. People with mild asymptomatic thrombocytopenia/subacute TTP episodes thus tend to defer or avoid the therapy, with long‐term effects including silent thrombotic organ failure, cognitive impairment, and depression.^28^ The arrival of recombinant ADAMTS‐13 (now in phase III)^29^ will allow regular and possibly home treatment, but availability may be limited and cost implications significant, indicating a need for further/adjunct options. Additional tools for overcoming the consequences of losing ADAMTS‐13 activity could therefore be of benefit.

Statins are drugs designed to lower cholesterol by inhibiting 3‐Hydroxy‐3‐methylglutaryl‐coenzyme A reductase, but that also have pleiotropic beneficial effects,^19^, ^30^ leading to a reduced risk of cardiovascular disease and thrombosis and a decrease in all‐cause mortality,^31^ yet without causing bleeding.^32^ In addition to these effects, statins also unlink the stacks of cisternae forming the Golgi ribbon, where WPBs are formed, thus enforcing the formation of shorter WPB organelles without affecting multimerization of VWF.^9^ The shorter WPB reduce the ability of agonist‐driven released VWF to recruit plasma VWF and platelets,^10^ thereby supporting the reported antithrombotic effect of statins, which include, but are not limited to, increase in nitric oxide production, and decrease in tissue factor and plasminogen activator inhibitor 1.^33^, ^34^, ^35^

We tested the ability of fluvastatin‐treated endothelial cells to recruit plasma VWF and platelets in the presence of either ADAMTS‐13–rich control plasma or ADAMTS‐13–poor plasma from patients with cTTP. We used a simple in vitro flow system to determine the specific effect of plasma origin, while excluding other additional contributors such as the effects of statins on platelet aggregation. The addition of identical platelets (from healthy donors), to platelet‐poor plasma from healthy patients or patients with TTP immediately before the assay, provides a clear snapshot of the consequences of treating an endothelial monolayer with fluvastatin. In agonist‐activated untreated cells, the total cumulative length of VWF strings is much higher when TTP plasma is superfused, as already reported,^8^ but in fluvastatin‐treated cells we observe a dramatic decrease in both string length and number, reflected in a decrease in recruitment of plasma VWF, that altogether cause a considerable reduction in platelet adhesion, not only in the presence of control but also, and more dramatically, in the presence of patient plasma.

The data suggest that there may be benefits for patients with cTTP in using fluvastatin as an additional prophylactic to reduce the risk of acute episodes and of nonovert symptoms.^21^ Among the potential advantages of this particular drug is that statins are considered safe and their effect on WPBs (at least in vitro) is fast (24 hours), compared to their effects in reducing cholesterol levels, which require significantly longer to stabilize.^36^ Indeed, statins have been successfully exploited perioperatively.^20^, ^37^, ^38^ These in vitro proof‐of‐principle studies using an experimental model provide a foundation from which the concept could be expanded to in vivo models and then to clinical trials.

We previously identified statins as WPB‐shortening drugs,^10^ and showed that they diminish formation of the long platelet‐recruiting VWF strings that form after exocytosis and reduce recruitment of plasma VWF to the endothelial surface. We here take this further by now showing that fluvastatin’s WPB‐mediated antithrombotic effect can also entirely overcome the enormously detrimental amplifying effect on these two aspects of hemostatic initiation occurring when using plasma samples of patients with cTTP. These data suggest a potential antithrombotic with an entirely different mode of action. While progress of this work requires a clinical study, we provide not only a rationale supporting an additional therapy for cTTP but also a supplementary rationale for the mode of action of this drug in more common cerebrovascular disorders.

## RELATIONSHIP DISCLOSURE

DC, FP, OC, and CV disclose no conflicts of interest. MAS discloses speaker’s fees and advisory boards: Takeda, Alexion, Sanofi, Novartis, Octapharma.

## AUTHOR CONTRIBUTIONS

Conceptualization: DFC, MAS, and FP designed the research; methodology: DFC, FP, and MAS; validation: FP and CV; formal analysis: DFC, FP, and CV; investigation: FP, CV, and OC; data curation: FP; writing—original draft: DFC and FP; writing—review and editing: DFC,FP,CV,and MAS; visualization: FP; supervision: DFC and MAS; project administration: DFC and MAS; funding acquisition: DFC and MAS.

## Supporting information

Fig S1

Fig S2

Supplementary Material

## Data Availability

For data, please contact Dan Cutler, d.cutler@ucl.ac.uk.
