# Supplementary figures and images for: Shrinking Weibel‐Palade bodies prevents high platelet recruitment in assays using thrombotic thrombocytopenic purpura plasma
Source: Res Pract Thromb Haemost. 2021 Dec 7;5(8):e12626. doi: 10.1002/rth2.12626 (PMC8652131; doi:10.1002/rth2.12626)

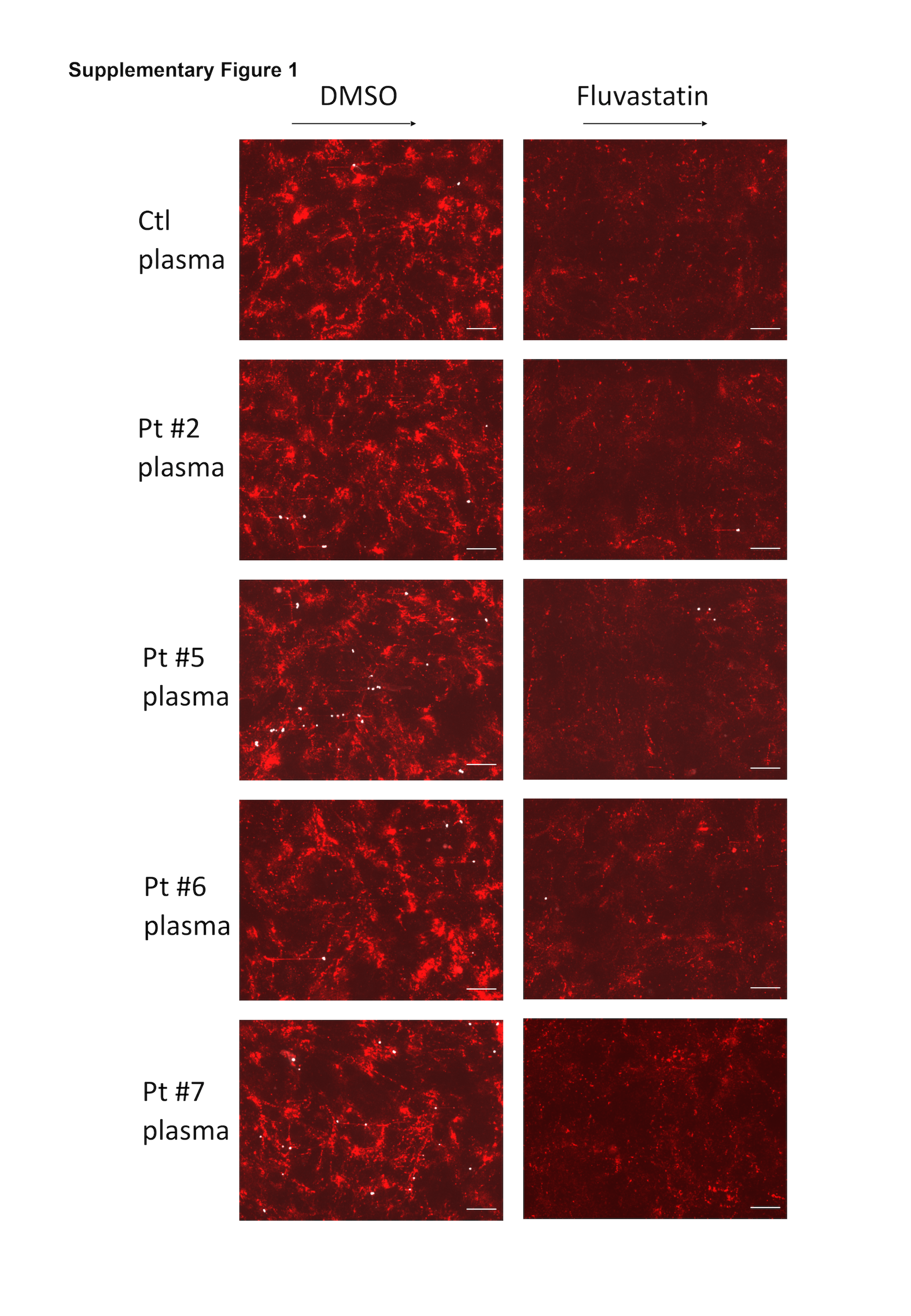

Supplement: Supplementary file 1 — Fig S1 [file RTH2-5-e12626-s003.tif]

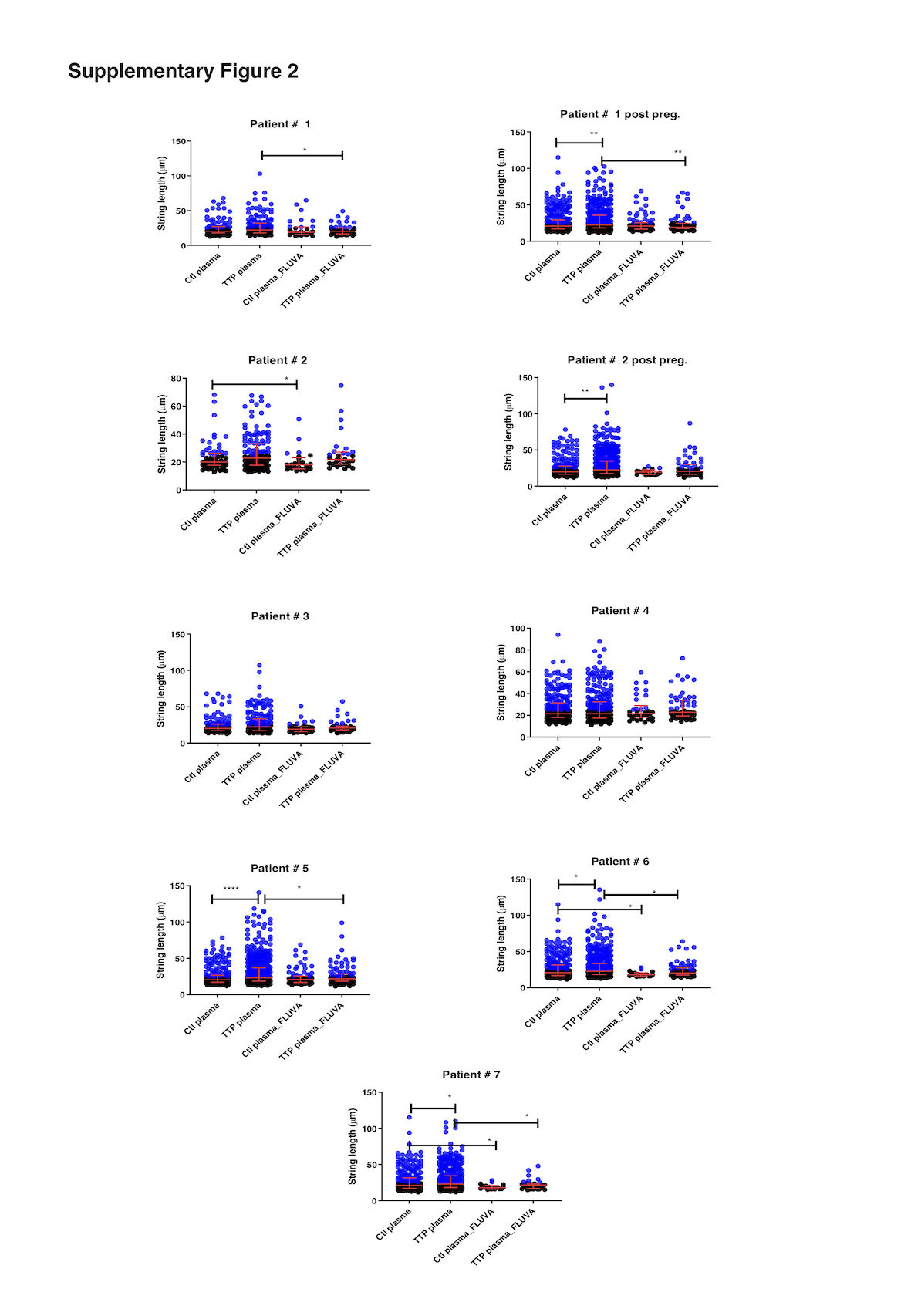

Supplement: Supplementary file 2 — Fig S2 [file RTH2-5-e12626-s001.tiff]
