# Supplementary material for: Shrinking Weibel‐Palade bodies prevents high platelet recruitment in assays using thrombotic thrombocytopenic purpura plasma
Source: Res Pract Thromb Haemost. 2021 Dec 7;5(8):e12626. doi: 10.1002/rth2.12626 (PMC8652131; doi:10.1002/rth2.12626)
Supplement: Supplementary file 3 — Supplementary Material [file RTH2-5-e12626-s002.docx]

**Supplementary Figure 1. Immunofluorescence images of endothelial cells pre-treated with fluvastatin (or DMSO) and subjected to flow assay with plasma and platelets.** Endothelial seeded in Ibidi μ-slides were treated or not with 2uM Fluvastatin for 24h. The slides were then connected to a pump providing a constant wall shear stress of 2.5 dynes/cm^2^ (corresponding to a flow rate of 1.4 ml/min). After a wash with buffer, the cells were stimulated with histamine and then superfused with plasma from pooled controls or individual TTP patients. After fixation the slides were disconnected from the pump and immunofluorescence was performed and confocal tiled images were taken. The arrows indicate the direction of the flow. Red= VWF. VWF strings are visible as a linear structures aligned with the direction of the flow, sometimes decorated with platelets. Grey= platelets (CD41). Scale bar= 50 µm.

**Supplementary Figure 2. Distribution of string lengths per patient.**

Each graph includes the string length measurements of 3 replicate experiments pooled together.

Strings longer than 25 µm are shown in blue. Non param. Mann-Whitney test, **p<0.005, *p<0.05. Median and interquartile range are shown.
